# The influence of the grass mixture composition on the quality and suitability for football pitches

**DOI:** 10.1038/s41598-021-99859-9

**Published:** 2021-10-18

**Authors:** Karol Wolski, Joanna Markowska, Adam Radkowski, Marek Brennensthul, Łukasz Sobol, Grzegorz Pęczkowski, Henryk Bujak, Wiktoria Grzebieniarz, Iwona Radkowska, Karen Khachatryan

**Affiliations:** 1 Institute of Agroecology and Plant Production, Wroclaw University of Environmental and Life Sciences, Wrocław, Poland; 2 Institute of Environmental Engineering, Wroclaw University of Environmental and Life Sciences, Wrocław, Poland; 3 Department of Agroecology and Plant Production, University of Agriculture in Kraków, Kraków, Poland; 4 Institute of Agricultural Engineering, Wroclaw University of Environmental and Life Sciences, Wrocław, Poland; 5 Institute of Environmental Protection and Management, Wroclaw University of Environmental and Life Sciences, Wrocław, Poland; 6 Department of Genetics, Plant Breeding and Seed Production, Wrocław University of Environmental and Life Sciences, Wrocław, Poland; 7 Research Centre for Cultivar Testing in Slupia Wielka, 63-022, Slupia Wielka, Poland; 8 Faculty of Food Technology, University of Agriculture in Krakow, Balicka Str. 122, 30-149 Kraków, Poland; 9 Department of Cattle Breeding, National Research Institute of Animal Production, Krakowska 1, 32-083 Balice, Poland

**Keywords:** Plant sciences, Environmental sciences

## Abstract

The selection of grass mixtures with appropriate visual and functional parameters for sowing football fields is a key element in shaping the sports infrastructure, ensuring the spectacularity of a match and comfort for players. The aim of the research was to investigate the properties of lawn grass mixtures and their suitability for football pitches. The experiment was conducted at the Toya Golf & Country Club (51° 20′ E, 17° 07′ N), Wrocław, Poland, between 2007 and 2009. 12 grass mixtures were selected, mainly based on red fescue, Kentucky bluegrass, and perennial ryegrass. The assessment was carried out using a nine-point scale, according to the Plant Variety Office methodology for crops and turf grass. Six features of sports turf were studied: appearance, density, colour, leaf fineness, overwintering, and susceptibility to disease and they significantly varied, depending on the grass mixture and the year of research. Our study showed that mixtures based on the dominance of meadow grass were characterized by higher values of the general visual aspect, colour and slenderness of the leaf blade and these based on the dominance of perennial ryegrass and co-dominance of perennial ryegrass and meadow grass were the most useful in terms of wintering, resistance to diseases and sodding.

## Introduction

The selection of proper grass species and varieties is considered to be one of the most important elements in the installation of sports pitches^1^. Well selected lawn grass mixtures ensure the best functional and visual parameters of the turf^2^. Intensive use of sports turf contributes to an increase in grass exposure to stress factors^3^. Therefore, it is appropriate to use a few species and varieties of lawn grass, instead of one, since this results in an increase in the resistance of sports turf to diseases, pests, and unfavourable weather conditions^4^. In addition, proper installation of sports turf contributes significantly to a more spectacular game^5^. Thomson and Rennie^6^ report that progress in the preparation and maintenance of grass surfaces has significantly accelerated the evolution of the game, allowing players to perform more matches, sprint rapidly, and move more than ever before^7^. It should be noted that, the appropriate condition of the sports turf guarantees the attractiveness of the event in visual and functional terms^8^, with better conditions for bouncing, rolling, and running after the ball^9^. It is also worth noting that the correct selection of lawn grass mixtures can also significantly contribute to reducing the risk of injuries on sports fields^10^. There are differences between grass species broad enough to select appropriate composition of a mixture^11^, otherwise the functional value of a football pitch may be lower. It is reported that in the warm climate, the best grasses for sports turf are Cynodon dactylon, Cynodon transvaalensis^12^, and Zoysia spp.^13^. In the temperate climate zone, the most common species include *Lolium perenne *L., *Poa pratensis *L., *Festuca rubra *L. and *Festuca arundinacea Schreb*^14^. Unfortunately, despite the wide variety of grass species and commercial grass mixtures available, they are often sown incorrectly, which significantly reduces the quality of sports fields.

Usually, for financial reasons, a lot of commercially available grass mixtures are imported, even if they are often not adapted to local climatic conditions^15^. In addition, different grass species are often mixed in order to reduce the financial outlays related to the maintenance of sports fields. Such action reduces energy and water consumption during mowing or turf irrigation^10^. However, it may lead to a decrease in the functional and visual parameters of the turf, as a result of which its playability may deteriorate. It is also worth noting that at present, in the mixtures of lawn grasses in the temperate climate is limited and strong dominance of *Festuca rubra* is noticeable. For this reason, alternative, competitive solutions are currently being sought that will allow the above-mentioned effects to be achieved, while maintaining high-quality turf, ensuring appropriate conditions for the game. One way to achieve this goal is to develop and test new species and types of lawn grasses, and to mix them together^16^. Potentially discovered mixtures may allow the required visual and functional standards to be met, therefore make a significant contribution to the installation of well-functioning natural sports turf in temperate climate conditions. In order to determine the suitability of new grass mixtures for sowing football and sports fields, it is necessary to perform a comprehensive evaluation of tests, including the functional evaluation of sports turf^17^. The experiments are most often carried out according to the methodology of Lawn National Turfgrass Evaluation Program (NTEP) and the Research Centre for Cultivar Testing (COBORU, Poland) (Domański, 1998; Polish DIN Standard 18035-4, n.d.). The research is conducted based on the valuation (Domański, 1998) and visual method (Turgeon 2004).

During the turf testing with the valuation method, particular emphasis should be placed on the aspects related to the general appearance of the turf, the density of the turf and the color of the grasslands^17^. Comprehensive assessment of these parameters is made by assigning points to the turf, using a 9-point scale. During the valorization of turf with the visual method, two basic parameters are usually assessed: the number of shoots per cm^2^ and the texture of the turf—the width of the leaf blade. The rules for assessing these aspects are the same as for the valuation method. However, it should be noted that the evaluation of lawn grass mixtures is a subjective process, based on visual assessments of the above factors. According to NTEP, this is dictated by the fact that the parameterization of the quality of sports grasses does not directly relate to the yield or nutritional value, therefore these factors cannot be assessed in a similar way to agricultural crops. The quality of the sports turf should therefore be included in the measure of aesthetics, which is more complex and difficult to assess (A Guide to NTEP Turfgrass Ratings)^18^.

It is also worth emphasizing that during the evaluation of the quality and suitability of the turf for sports purposes, other tests are carried out, which are related primarily to the functionality of the turf—e.g. measuring the depth of the range of the main root mass, or grass propagation^19^. Important parameters in relation to the quality of football turf are also Surface Mechanical Testing^20^, including: (i) ball-surface interaction; (ii) surface performance and aesthetics; and (iii) player-surface, wintering and grassland resistance to pathogens^21,22^.

As mentioned earlier, in commercial mixtures dedicated to sports fields, we can notice a strong dominance of mixtures based mainly on *Festuca rubra* L. This is due to the fact that this species is considered one of the most popular for sowing sports fields located in Europe and North America^23^.

A smaller number of commercial mixtures are based on *Lolium perenne* and *Poa pratensis* varieties, which can also be successfully used to sow sports fields in various climatic conditions^24^.

However, there is still little information in the literature about the quality of the turf based on these two grass species. In recent years, more items evaluating ryegrass and bluegrass pitches have become available, but they did not include a wide range of grass varieties or did not consider different shares of the same varieties in the mixture composition^25–28^. According to Friell et al., grass mixtures characterized by similar species composition but different percentage of species or different varieties of the same species, may significantly differ from each other and react to stress completely differently^29^. Hence, it should be recognized that there is a high need for a wide evaluation of grass mixtures based on the dominance of perennial ryegrass and common panicle, with a diverse composition of varieties of both species and the percentage composition.

The aim of the research was to determine the functional value of grass mixtures used to install football pitches. The studies were designed based on the hypothesis H1.

H1: The grasslands sown with composed lawn grass mixtures based on perennial ryegrass and Smooth-stalked Meadowgrass—*Poa pratensis* L. are of higher quality and usefulness for football fields compared to commercial grass mixtures based on red fescue.

## Materials and methods

### Study site

The experiment was conducted at the Toya Golf & Country Club (51° 20′ E, 17° 07′ N) between 2007 and 2009. The experiment was conducted on anthropogenic soil, the order of culture-earth soil, the type of hortistol, developed from loamy sand. Its granulometric structure was suitable for setting up natural football turf. Topsoil consisted mostly of coarse and medium-grained sand of 0.1–1 mm (about 70%). The silt fraction (0.1–0.02 mm) did not exceed 17%. The smallest fraction (0 > 0.02 mm) was 2% higher than Polish DIN Standard 18035-4 (Polish DIN Standard 18035-4). In the first and last year of the experiment (2007 and 2009), pH in topsoil ranged from 6.8 to 7.0. During the study period, weather conditions varied relatively little (Table 1). The highest dissimilarity was recorded in the last year of research (2009), when the total rainfall was 751.9 mm, with 598.1 mm in 2007 and 549.7 mm in 2008. Additionally, the same year proved to be the coldest of all, with its average air temperature of 9.3 °C (below zero in January and December), while in the remaining years it did not vary much and ranged between 10.2 and 10.3 °C.

**Table 1:** Monthly precipitation and average daily air temperature at the experiment site from 2007 to 2009.

Month	Rainfall, mm				Mean temperature, °C			
	2007	2008	2009	Average	2007	2008	2009	Average
January	52.0	56.7	34.6	47.8	4.9	2.9	− 2.3	1.8
February	59.0	20.4	46.4	41.9	2.7	3.9	0.2	2.3
March	48.8	33.0	49.5	43.8	6.5	4.5	4.6	5.2
April	2.7	87.1	30.9	40.2	10.9	8.9	12.0	10.6
May	50.3	37.3	67.5	51.7	15.6	14.3	14.2	14.7
June	69.2	36.5	162.0	89.2	19.2	18.8	15.8	17.9
July	120.6	65.6	134.2	106.8	19.2	19.9	19.5	19.5
August	52.8	94.0	53.5	66.8	18.9	18.8	19.3	19.0
September	46.1	27.9	12.0	28.7	12.9	13.2	14.8	13.6
October	21.7	41.1	76.0	46.3	8.3	9.6	7.9	8.6
November	53.9	29.6	32.5	38.7	2.8	6.1	6.6	5.2
December	21.0	32.5	51.9	35.1	1.0	2.1	− 0.4	0.9
Total/mean	598.1	549.7	751.0	636.9	10.2	10.3	9.3	9.9

### Experimental setup

The experiment was conducted on micro-plots of 1 m^2^, with the total area of 72 m^2^. It was founded in the spring of 2007, with a split-plot design, two variables, and three replications. The first variable (A) was the study year and the other (B) was M1–M12 grass mixtures. Seeds of grass mixtures were sown by hand, then, the plots were raked and a smooth roller was applied. In the experiment 12 grass mixtures were used (Table 2). Mixtures M1–M5 were available in the commercial offer, with the predominating varieties of red fescue in the species composition. Mixtures M6–M12, in which *Lolium perenne* L. and *Poa pratensis* L. varieties dominated, were purposefully prepared in the Department of Meadows and Green Area Creation in Wrocław University.

**Table 2:** Species composition of lawn grass mixtures.

Mixture	Latin name		Variety	Share %
M_1_	*F. rubra*		Adio + Leo + Mirena	20 + 20 + 20
	*L. perenne*		Gazon	20
	*P. pratensis*		Miracle	15
	*A. capillaris*		Kita	5
M_2_	*F. rubra*		Mirena + Leo + Adio	30 + 20 + 20
	*L. perenne*		Natara	30
M_3_	*F. rubra*		Areta + Adio	40 + 30
	*L. perenne*		Stadion	20
	*P. pratensis*		Miracle	10
M_4_	*F. rubra*		Adio + Leo + Mmirena	25 + 25 + 15
	*L. perenne*		Stadion	25
	*P. pratensis*		Miracle	10
M_5_	*F. rubra*		Leo + Aareta	50 + 20
	*F. arundinacea*		Asterix	20
	*P. pratensis*		Alicja	10
M_6_	*L. perenne*		Barball + Bardorado	25 + 25
	*P. pratensis*		Bariris + Miracle	25 + 25
M_7_	*P. pratensis*		Bariris + Miracle	35 + 35
	*L. perenne*		Barball + Bardorado	15 + 15
M_8_	*P. pratensis*		Bariris + Miracle	40 + 40
	*L. perenne*		Barball + Bardorado	10 + 10
M_9_	*P. pratensis*		Bariris + Miracle	20 + 20
	*L. perenne*		Barball + Bardorado	20 + 20
	*F. rubra*		Barcrown + Barustic	10 + 10
M_10_	*P. pratensis*		Bariris + Miracle	25 + 25
	*L. perenne*		Barball + Bardorado	20 + 20
	*F. rubra*		Barcrown + Barustic	5 + 5
M_11_	*L. perenne*		Barball + Bardorado	40 + 40
	*P. pratensis*		Bariris + Miracle	10 + 10
M_12_	*L. perenne*		Barball + Bardorado	20 + 20
	*P. pratensis*		Bariris + Miracle	20 + 20
	*F. arundinacea*		Asterix	10
	*F. rubra*		Barcrown + Barustic	5 + 5

The first mowing was done when the grass was 8 cm tall, and subsequent ones were carried out 1–2 times a week at a height of 3 cm. Turf, depending on the weather, was irrigated with 6 L m^−2^ of water per day. During each growing period, NPK mineral fertilizers were used in a ratio of 6:2:4, at the following doses: N—180 kg ha^−1^; P—60 kg ha^−1^; K—120 kg ha^−1^. Fertilizers were applied from April to September (Table 3), using Professional Spring–Summer (Hortnas Ltd., Góra, Poland) with an NPK ratio of 17–6–11 + MgO + S + B and Professional Autumn with an NPK ratio of 5–0–25 + S + Ca + Fe + B.

**Table 3:** Schedule of mineral fertilizer application.

Month	Quantity kg ha^−1^		
	N	P	K
April	45	30	30
May	30	0	15
June	45	30	15
July	30	0	15
August	30	0	15
September	0	0	30
Total	180	60	120

### Assessment criteria

Observations and measurements of grass mixtures were made three times a year: in the spring (April/May), summer (early August), and autumn (early October). The functional value of football pitches was assessed according to the Plant Variety Office methodology^30^ for arable crops and lawn grass. The results of the observations were recorded using a nine-point scale (from 1 to 9). The points indicated the rating of a grass feature, with 9 being the most favourable and 1 the least. Six selected features of football pitches were assessed: overall aspect, density, colour, susceptibility to disease, leaf fineness, and overwintering.

Overall aspect, which is a synthetic assessment of turf appearance, was rated using the following scale: (1) bad (no plants); (2) bad to weak; (3) weak (unattractive turf); (4) weak to sufficient; (5) sufficient (medium-quality turf); (6) sufficient to good; (7) good (looking nice); (8) good to very good; (9) very good (very attractive turf).

Density, which is a degree of ground coverage with grass stems and leaves, was assessed using the following scale: (1) bad (no plants) 0–5%; (2) bad to weak—6–15%; (3) weak (sparse grass)—16–25%; (4) weak to sufficient—26–40%; (5) sufficient (medium density)—41–60%; (6) sufficient to good—61–75%; (7) good (small grassless patches)—76–85%; (8) good to very good—86–95%; (9) very good (very dense grass)—96–100%.

Colour was assessed using catalogue numbers of the Royal Horticultural Society Colour Chart, Edition V^31^, with the following scale: (1) yellowish green—no. 144 A, B, C, D; (2) olive green—no. 138 A, B, C, D and 137 A, B, C, D; (3) bright green—no. 134 A, B, C, D; (4) greyish green—no. 133 A, B, C, D; (5) vivid green—no. 132 A, B, C, D; (6) green—no. 131 A, B, C, D; (7) grassy green—no. 135 A, B, C, D; (8) brownish green—no. 136 A, B, C, D; (9) emerald—no. 127 A, B, C, D.

Susceptibility to disease was assessed by determining the degree of plant infection by diseases (mainly snow mould, whitefly, pink patch, rust, and others) using the following scale: (1) very large (plants completely destroyed); (2) very large to large; (3) large (epiphytotic disease); (4) large to medium; (5) medium (only some plants are infected or destroyed); (6) medium to small; (7) small (few plants infected); (8) small to very small; (9) very small (no symptoms).

Leaf fineness was determined by assessing the leaf blade with its width and thickness, according to the following scale: (1) very wide (coarse); (2) coarse to wide; (3) wide; (4) wide to medium; (5) medium (typical); (6) medium to narrow; (7) narrow (slender); (8) narrow to very narrow; (9) very narrow (long and thin).

Overwintering was determined by comparing ground coverage by live plants in the autumn and in the spring 14 days after the growing period begins, according to the following scale: (1) very bad (86–100% of plants lost); (2) very bad to bad (76–85%); (3) bad (61–75%); (4) bad to medium (46–60%); (5) medium(36–45%); (6) medium to good (26–35%); (7) good (16–25%); (8) good to very good (6–15%); (9) very good (0–5% of dead plants).

### Statistical analysis

Firstly, the normality of distribution of the observed traits was tested using the Shapiro–Wilk normality test. Non-normal traits were transformed using the power (Box–Cox) transformation with lambda (λ) parameter at interval from − 2 to 2. Having the variables transformed and normally distributed, it was assumed that the data followed the multivariate normal distribution. The statistical analyses such as three-way (year, seeding rate, grass mixtures) analysis of variance (ANOVA), Tukey’s honestly significant difference (HSD)test for comparisons of pairs of means^32^ were performed according to the model of data obtained from the experiment designed as a split-plot. All calculations were carried out using GenStat v. 18 software package. Statistical significance was defined at 0.05 level depending on the source of variation. The results are presented in the Supplementary Data.

### Ethical statement

The collection of plant material (grass species used in the mixtures: *F. rubra, L. perenne, P. pratensis, A. capillaris, F. arundinacea*) complies with institutional, national, and international guidelines and legislation. The aerated grass used to establish the experiment was obtained from the Plat Breeding and Acclimatization Institute National Centre for Plant Genetic Pesources: Polish Genebank Radzików, 05-870 Błonie, Poland (EGISET—database system for documentation of NCPGR collections was implemented in 2010) and was approved for use for research purposes.

## Results

### Overall visual aspect

**Figure 1:**
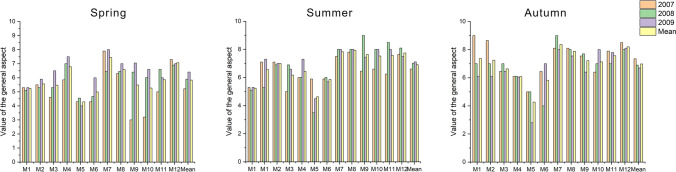
Ratings of pitch overall aspect across grass mixtures and research years.

Generally ratings of turf visual quality varied over the years of research (Fig. 1). During the spring, the average values for all lawns increased in consecutive years (2007—5.15, 2008—5.90, 2009—6.40), and for most mixtures (8 out of 12), the highest ratings were recorded in the spring of the last year (2009). The turf rated as the least attractive in 2007 was the M9 mixture with perennial ryegrass and Kentucky bluegrass. Its appearance was rated as 2.99, which corresponded to ‘bad to weak’. On the other hand, the highest visual value was recorded in 2009, when the bluegrass M7 turf, as the only one among the mixtures, was rated as ‘good to very good’ (8.01). Additionally, the same lawn proved to be attractive across all spring seasons even if the visual value of the overall aspect was slightly lower than the above and amounted to 7.45, defined as ‘good’ (looking nice). It is worth noting that the M12 multispecies turf was in the same range, but with a bit lower score (7.08). By far the least attractive lawn was that with the M5 fescue mixture, as the only one assessed as ‘weak to sufficient’ (4.28). The average spring ratings of the remaining mixtures ranged from 5.02 to 6.76.

Summer, as with spring assessment, it was observed that the attractiveness of the turf increased in consecutive years. In 2007 and 2008, the lawns were described as ‘sufficient to good’, receiving the ratings of 6.60 and 6.92, respectively. In the last year of the experiment, their visual value increased to ‘good’ (7.08). The most attractive turf of all summer seasons was in 2008. It was then that the M9 multispecies mixture was rated as ‘very good’, receiving the highest score (9.00). In the same year, the worst pitch was that with the M5 fescue dominating mixture, described as ‘weak’ (3.50). The same lawn was of the lowest visual value as the average of the summer seasons, but with a higher rating of ‘weak to sufficient’ (4.54). On the other hand, the best looking turf was that with the M8 bluegrass mixture, rated as ‘good’ (7.90). It is worth noting, however, that as many as six other lawns (M2, M7, M9, M10, M11, M12) also received similar ratings, even if a bit lower, ranging from 7.02 to 7.84.

During the autumn season, in contrast to the spring and summer, there was an opposite with the overall visual aspect ratings declining in consecutive years. In the first year of the experiment (2007), the lawns were defined as ‘good’ (7.34), while in the subsequent years they were assessed lower (in 2008—6.86, in 2009–6.60). During the experiment, two mixtures were rated as very attractive, with the highest score (9.00). The first was M1 with red fescue, in the first year of the experiment (2007), and the other was M7 with bluegrass, in the second year. The least decorative turf was the M5 fescue mixture in the last year (2009), with its autumn appearance rated as ‘bad to weak’ (2.76). On average, throughout the experiment, this mixture was also of the lowest visual value. In the autumn of the final year, the turf was rated as ‘weak to sufficient’ (4.20), but in the first two years the rating was higher (5.02). On the other hand, the M7 bluegrass mixture was rated the highest, ‘good to very good’ (8.41). It should also be noted that the M12 multispecies mixture was rated in the same range, but with a slightly lower score (8.24).

### Density

**Figure 2:**
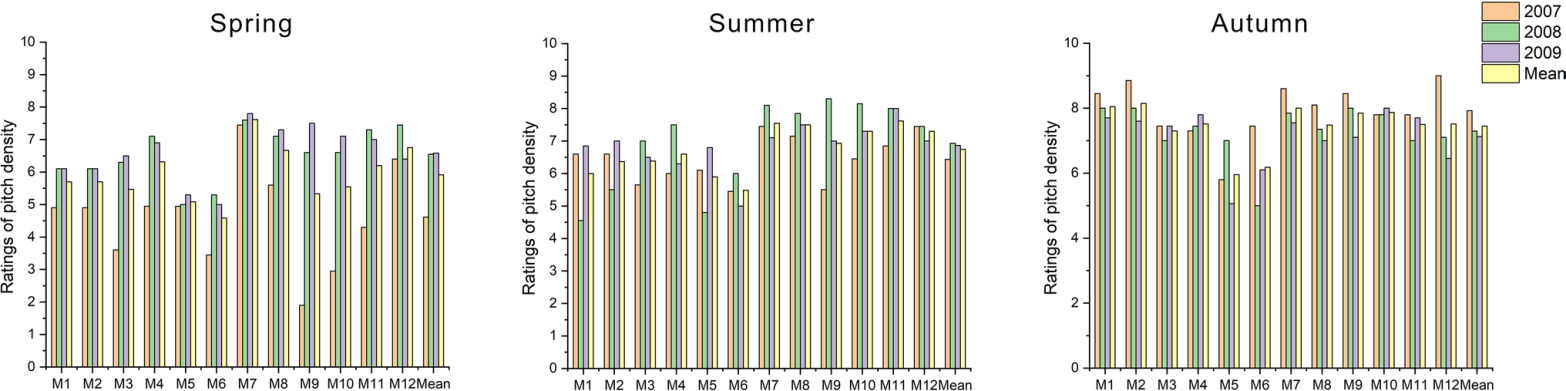
Ratings of pitch density across grass mixtures and research years.

Significant differences density ratings of grass mixtures were recorded during all seasons (Fig. 2). In the course of spring it was observed that in the year of experiment instalment (2007) it was weaker than in subsequent years. The exception was the M12 multispecies turf, which in the first and last year reached the same value range, rated as ‘good’ (6.50). The worst density in the spring of that year (2007) was recorded for the M9 turf, with a dominating share of perennial ryegrass and Kentucky bluegrass, and M10, with Kentucky bluegrass, with both scoring the lowest rating. Their density was assessed, respectively, as ‘bad’ (1.96) and ‘bad to weak’ (2.96). It is worth noting, however, that the appearances of the M9 turf was significantly more attractive (7.67) in the last year of the experiment (2009). In the same year in the spring a higher value was recorded only for the bluegrass M7 turf, rated as ‘good’ (7.84). Additionally, among all grass mixtures M7 turf density was rated the highest (7.67). It should be emphasized that already in the first year, it was assessed as ‘good to very good’ (7.51). On the other hand, the worst turf during the spring turned out to be that with the M6 two-species mixture (50% *L. perenne*, 50% *P. pratensis*), rated as ‘sufficient’ (4.58).

However, during summer season, no tendency was noticed similar to that in the spring, when the turf in the first year was assessed lower than in subsequent years. Admittedly, this rising tendency was also observed in the summer, but it was not as pronounced as for the spring. On average, the turf was of the best density in the second year of the experiment (2008), when the highest and lowest values among the lawn mixtures were recorded; the M9 turf was rated the highest, as ‘good to very good’ (8.35), while the M1 fescue lawn, whose turf was described as ‘weak to sufficient’, was of the lowest density (4.67). On average, during the summer, the worst turf, as in the spring, turned out to be on the M6 two-species lawn, rated as ‘sufficient’ (5.52). On the other hand, the bluegrass M7 turf and the ryegrass M11 turf had both the best density, with their average score of 7.62, rated as ‘good’.

Similar conclusions were recorded in the autumn. In the first year, on eight lawns the values of the parameter were higher than in subsequent years, which was different from the trend recorded in the spring and summer. On average, in 2007 the density rating was ‘good’ (7.95), while in the subsequent years it was lower (for 2008—7.29, for 2009—7.13). The highest value in 2007 was assigned to the M12 four-species mixture (40% *L. perenne*, 40% *P. pratensis*, 10% *F. rubra*, 10% *F. arundinacea*), the density of which was ‘very good’ (9.00). On the other hand, the worst density (5.02) was recorded in 2008 for the perennial ryegrass and Kentucky bluegrass mixture (M6), with a species composition of 50% of *L. perenne* and 50% of *P. pratensis*. On average, during the autumn seasons, the most favourable density was on the turf with the M2 fescue mixture, the value of which was 8.18. It is also worth noting that the M1, M7, M9, and M10 mixtures, whose density rating was in the range of 8.07–7.90, were also of relatively ‘good to very good’ density. By far, the worst turf (5.02) was formed by the two-species M6 mixture.

### Colour

**Figure 3:**
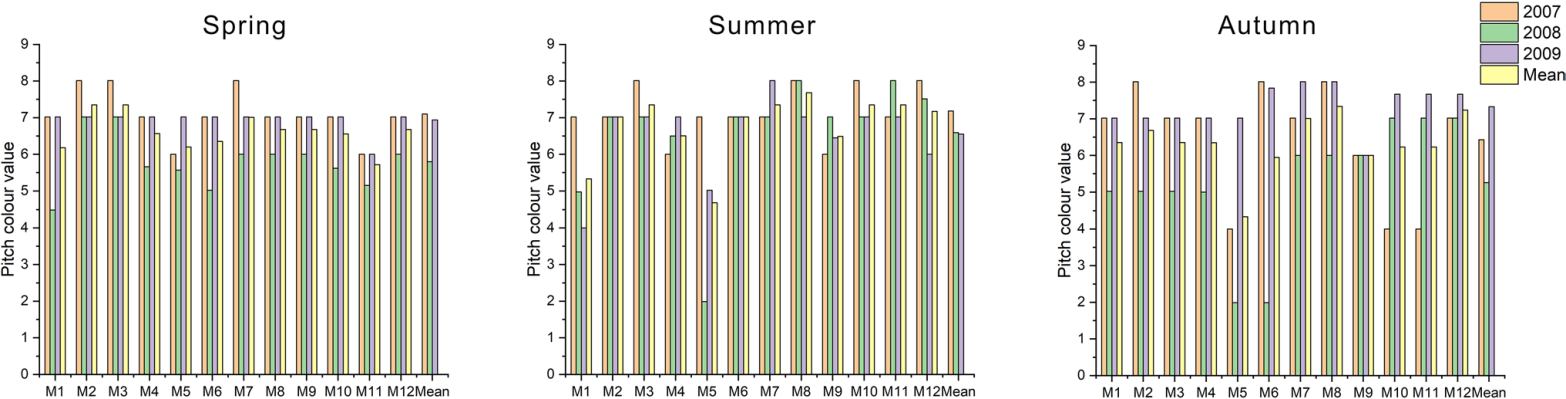
Ratings of pitch colour across grass mixtures and research years in different seasons.

Seasonally assessment of grass colour revealed significant differences across the years of research (Fig. 3). During the spring, there was no increasing or decreasing trend in consecutive years. However, in 2008, there was a decrease in the average colour attractiveness of all sports pitches, from ‘grassy green’ (7.02) in the previous year to ‘vivid-green’ (5.76). However, the value of the parameter a year later (2009) increased to that observed in the first year of the experiment (2007). In the spring, the darkest and most desirable colour (brownish green) was recorded on four lawns: three mixtures in the first year (2007), i.e. the M2 and M3 fescue pitches and the M7 bluegrass turf, and one in 2009, i.e. the M5 fescue turf. On the other hand, the least attractive colour (greyish green) was recorded in 2008 on the M1 fescue turf. On average, over the years, the most favourable colour in the spring (‘grassy green’) was assigned to the M2 and M3 fescue turf, assessed as 7.34. The bluegrass M7 turf was rated in the same range, but with a slightly lower value of 7.02. The lowest colour rating (5.71) was assigned to the ryegrass M11 turf.

During the summer, the best colour, ‘grassy green’ with 7.18 points, was recorded in the first year of the experiment (2007). In later years, there was a decrease in lawn attractiveness to ‘green’, from 6.45 points in 2008 to 6.50 in 2009. Throughout the experiment, the brownish green colour was recorded on seven lawns, each of them rated at 8.01. In the first year of research (2007) the turf of M3, M8, M10, M12 received this rating, the M8 and M11 turf in the second year (2008), and the bluegrass M7 mixture in the last year of the experiment (2009). The least attractive colour was noted in 2008 on the lawn with the M5 fescue mixture assessed as ‘yellowish green’ (1.99). The colour of the same turf, defined on average as ‘greyish green’ (4.41), was the least favourable across all summers. The colour of as many as eight pitches was assessed as ‘grassy green’. However, bluegrass M8 (7.67) was rated the highest. The values of seven remaining mixtures (M2, M3, M6, M7, M10, M11, M12) were in the range of 7.02–7.34.

In the course of autumn, as in the previous seasons, there was no downward or upward trend in consecutive years of research. The best ‘brownish green’ colour (8.01o) was noted five times, in 2007 on fescue M2, two-species M6, and bluegrass M8 lawns and in 2009 on bluegrass M7 and M8 pitches. The least favourable colour (‘yellowish green’) was recorded in 2008 on two lawns—the M5 fescue and the M6 two-species mixtures, both rated as 1.99. During the summer, the most attractive colour (‘grassy green’) was assigned to M8 and M12 turf, which obtained similar ratings (7.29 and 7.24, respectively). The least favourable colour was noted on the M5 fescue turf, whose rating was 4.08. The assessments of the remaining pitches ranged from 5.52 to 6.97, which corresponded to ‘vivid green’ and ‘green’.

### Leaf fineness

**Figure 4:**
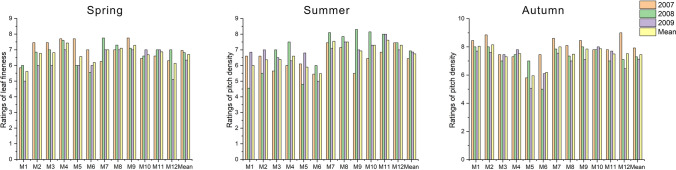
Ratings of leaf fineness across grass mixtures and research years.

Assessment of sports turf during the studies seasons revealed significant differences between leaf fineness ratings across the years of research (Fig. 4). There was a downward trend in the parameter in consecutive years, but the values remained within the 6.35–6.97 range, as ‘medium to narrow’. The most delicate and narrowest leaf blade was recorded in the second year of research (2008) on the fescue M4 turf. This lawn was assigned a rating of 7.84 during the spring. On the other hand, fescue M1 turf was assessed the lowest, rated as 5.02 in the last year. Both of the above-mentioned lawn grass mixtures were assigned extreme values. The M2 mixture produced turf with a ‘medium’ (typical) rating of the leaf blade, with a rate of 5.62. The M4 lawn, on the other hand, was rated the highest in the spring, with a ‘narrow’ (slender) leaf (7.51).

Summer, as in the spring, there was a downward trend in leaf fineness in consecutive years. The average value of the parameter changed from ‘narrow’ (7.45) in 2007 to ‘medium to narrow’ in subsequent years (6.86 in 2008 and 6.55 in 2009). The lowest value was recorded for the fescue M1 turf in 2009, with a rating of 5.02, which corresponds to a ‘medium’ (typical) leaf blade. The highest ratings were assigned four times to two mixtures, in 2009 to the M11 ryegrass turf, defined as ‘narrow to very narrow’ (8.01) and to the bluegrass M7 with the score of 8.01 in 2007, 2008, and 2009. The latter one was of the best leaf fineness throughout the experiment. On the other hand, the fescue M1 turf, rated as ‘medium to narrow’ (6.00), produced the least favourable leaves.

During the autumn, like for spring and summer assessment, the value of the parameter decreased in consecutive years. It fell from 6.86 in 2007 to 6.15 in 2009. The highest value was recorded in 2008 for two lawns with the M8 and M9 mixtures, producing ‘narrow to very narrow’ leaf blades. The lowest parameter value was recorded on two lawns, with 5.02 points for the M5 fescue lawn and the M6 two-species turf. Over the years, the bluegrass M8 turf produced leaves with the most desirable width and thickness, rated as 7.62. On the other hand, the M6 two-species turf was rated the lowest at 5.24. The average leaf fineness ratings of the remaining lawn grass mixtures was in the range of 5.71–7.34.

### Susceptibility to diseases

**Figure 5:**
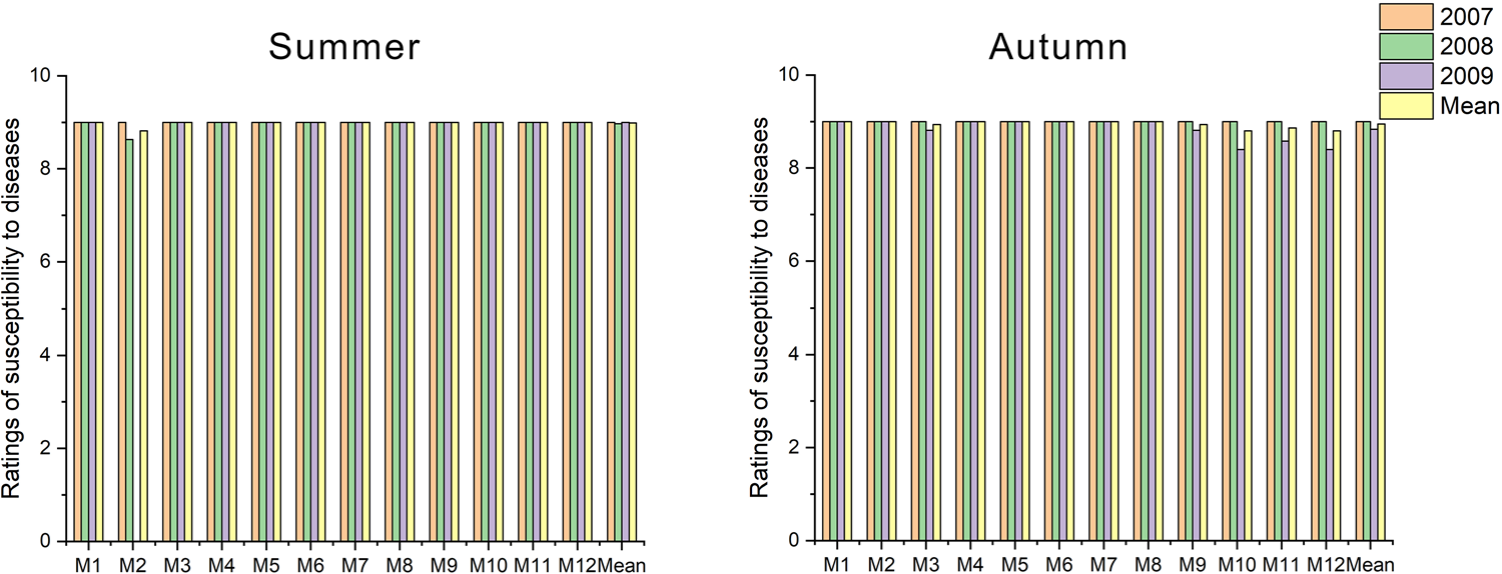
Ratings of susceptibility to diseases across grass mixtures and research years.

In the summer, in the first and second years of research (2007 and 2008) no signs of infection by fungal pathogens were observed on the lawns (Fig. 5). In the third year, there was a slight increase in the prevalence of fungal disease, pink patch caused by Limonomyces roseipellis. However, this was recorded only on the M2 fescue turf.

In the autumn, during the first two years of research, no signs of infection by fungal pathogens were observed either (Fig. 5). In the autumn last year (2009), as in the summer, the pathogen *Limonomyces roseipellis* was observed on the M3 fescue. However, the severity of the disease was low. During this period, single, scattered mushrooms grew up on the M9, M10, M11, and M12 turf.

### Overwintering

**Figure 6:**
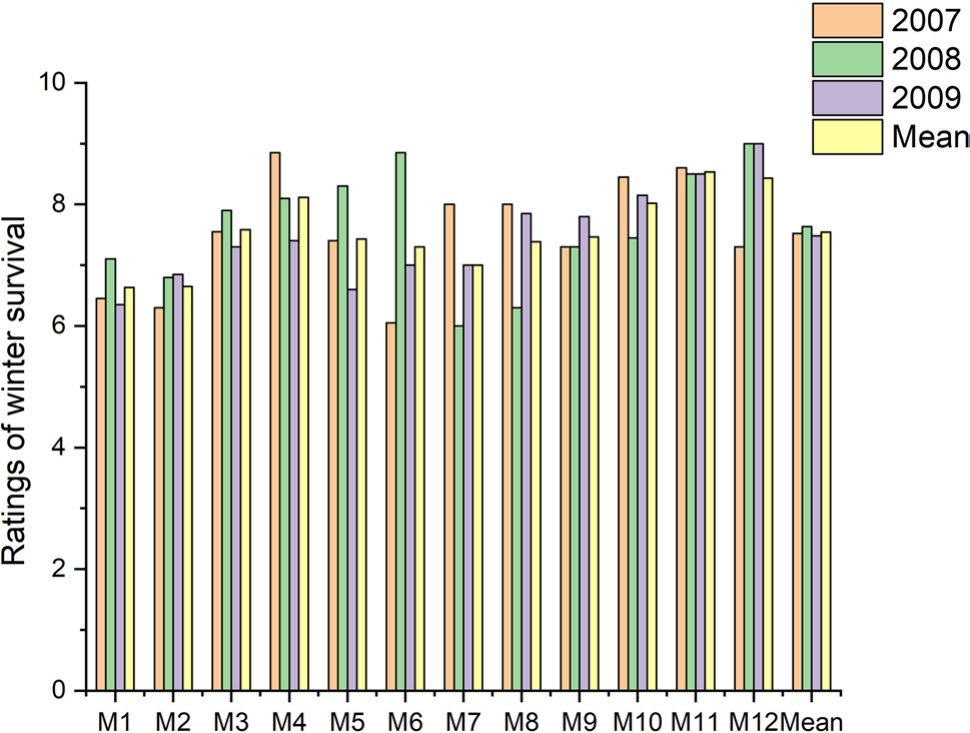
Ratings of winter survival across grass mixtures and research years.

The ratings of grass overwintering significantly varied over the years of research (Fig. 6). The highest values (7.62) were recorded in the second year (2008/2009), but in the other years they were slightly smaller, amounting to 7.56 after the 2007/2008 winter and to 7.51 after the 2009/2010 winter. The worst survival rate was recorded after the winter of 2008/2009 on the M12 lawn with the dominating share of perennial ryegrass and bluegrass, which received, on average, ‘medium to good’ rating (6.00). On the other hand, the best winter survival of the M12 turf was observed in the spring of 2009 and 2010, both times with the highest possible rating of 9.00. Over the years, the ryegrass M11 turf, whose overwintering rate was ‘good to very good’ (8.53), proved to be the most resistant to the cold. It is worth noting that the M12 turf (8.47) scored only a slightly lower number of points. On the other hand, the highest average percentage of dead plants (26%) were on the M1 and M2 fescue lawns, which both obtained the same ratings.

## Discussion

Visual quality assessment of sports pitches is conducted in many countries, among others in the United States and Europe, according to the Lawn National Turfgrass Evaluation Program (NTEP) and the Research Centre for Cultivar Testing (COBORU, Poland)^30,33^. The correct selection of lawn grass species and varieties is an important element in the installation of football pitches, making a sports event more involving. In the conducted experiment six selected characteristics of football pitches were examined—overall aspect, density, colour, leaf fineness, overwintering, and susceptibility to disease. The mixture components have significant impact on the visual quality of a sports pitch altogether with an interaction of their genotypes, and on environmental factors^34^. It was found that at different times of the year, the M7 and M8 pitches were of the most attractive visual quality. The species composition of these mixtures was dominated by Kentucky bluegrass, while the share of perennial ryegrass was smaller. Examining the visual value of registered and newly-created varieties of Kentucky bluegrass, Martyniak^35^ recorded a slightly lower average visual value ratings (6.9–7.0) than in the results obtained in discussed experiment. Grygierzec and Janus also recorded similar findings^36^, but depending on the variety, lawn appearance in their experiment was highly diverse. It is also worth noting that in many studies, only one variety of meadow bluegrass was used, with no mixtures with other varieties examined. In turn, Starczewski and Affek-Starczewska^37^ noticed that the greater the share of perennial ryegrass in the mixture, the higher its appearance was rated. Our findings and those of Jankowski et al.^38^ did not agree with these observations. Brede^39^ determined that that in bluegrass turf aesthetic value decreased during the seasons of the same year and throughout consecutive years of research, which was not confirmed in the present studies either. In the first two years of research, the attractiveness of the turf increased, whereas it decreased in the summer and autumn in the last year, which may be explained by the exceptionally low amount of rain in August and September (Table 1). Another assessed factor was density, i.e. ground coverage by grass leaf blades. The higher its value, the higher the ground coverage was^40,41^. Observations of the sports turf revealed a high variability in density over the years and seasons, depending on the grass mixture. The density of the lawn with the M7 bluegrass mixture in the spring and summer was rated the highest. Pornaro et al.^1^ also observed the highest density on a lawn with bluegrass, but the authors pointed out that density was significantly reduced under the conditions of the experiment. The results presented in this paper were also in concordance with findings of Jankowski et al.^42^, who claimed that, on average from years of research, lawn turf based on bluegrass was significantly more favourable. However, the literature did not present a unified opinion on the effects of different grass species on lawn density. Popovici et al.^43^ reported that the highest density was recorded for mixtures where the varieties of perennial ryegrass prevailed. In that experiment, however, the authors did not include mixtures in which Kentucky bluegrass would dominate the species composition. Starczewski and Affek-Starczewska^37^ noticed noticed that turf with dominating varieties of perennial ryegrass was the densest, but in the present experiment the highest average density was assigned to the M2 fescue pitch (70% of *F. rubra* and 30% of *L. perenne*) in the autumn. High ratings of the compactness of fescue varieties were confirmed by Radkowski and Stryc^40^. As in this paper, the authors, for most varieties, found that the most favourable density was in the autumn. Different results was recorded by Jankowski et al.^44^, in whose experiment the density value was rated significantly lower than in the present study. The next feature, grass colour, is considered a useful indicator of plant general condition. This parameter is defined as a visual perception of light reflected by the turf^8^. Visual assessment of grass colour is the best method of selecting suitable species and varieties to install a lawn^45^. Studies and observations of the turf showed high colour variability, depending on the grass mixture and the year of research. Comparable results were recorded by Jankowski et al.^42^, with a strong relationship between the colour of the turf and its species composition. The overall results of the experiment indicated that the most attractive turf colour was in the spring and summer, obtaining higher ratings, on average, by 0.35 and 0.46 higher than in the autumn. Our findings that the most attractive turf colour was in the spring and summer concurred with previous studies^46,47^. Grabowski et al.^48^ also noted a more downward trend in grass colour in the autumn than in other seasons. It should also be emphasized that in the second year, in each season, grass was of a less intense colour, which can be explained by worse weather conditions^23^. Observations and analysis of leaf fineness showed a strong variation in the width of the leaf blades depending on the grass mixture, season, and year. Two main types of mixtures with the highest values of the parameter were identified: the M4 fescue and the M7 and M8 both based on Kentucky bluegrass. The obtained results are in line with the literature data provided by Grabowski et al.^48^ which reported that red fescue produced much narrower leaf blades than other lawn grass species. Although the leaf fineness index among fescue mixtures did not vary much, the lawn with the M1 mixture was rated the lowest. The leaf blade of the mixtures with a dominating share of Kentucky bluegrass was usually assessed as narrow (7.00–8.00). In the literature, there were slightly lower rating of bluegrass leaf slenderness^49^. It is also worth noting that there was a clear downward trend in leaf fineness in consecutive years. Previous studies on the same aspect indicated that plants in the first year of research had wider leaf blades than in consecutive years, which was not observed in the present experiment. Another important feature of lawn grass mixtures assessed in the experiment was susceptibility to disease. This indicator is useful for installing lawns with disease resistant varieties and species, with no brown or bare patches of infected grass. Grass infected by disease loses its valuable aesthetic qualities. Currently, most diseases of turf grass are caused by fungi^50^, as confirmed in the present studies. However, the presence of these pathogens did not significantly affect other functional features of the turf. Pink patch observed on fescue turf is typical for this species, especially in the autumn and winter^51^. Similar observations have been made in other work—Prończuk^52^ indicates that fungi can appear even on properly cultivated and well-maintained lawn. The last feature to be assessed was overwintering. The ratings of the parameter over the years did not change significantly and ranged from 7.51 to 7.62. The M11 perennial ryegrass turf and M12 lawn, with Kentucky bluegrass and perennial ryegrass as dominating species, had the best winter survival rate. The results of the present experiment were reflected in the literature. Studying the effect of hydrogel, Jankowski et al.^53^ found that it raised the overwintering rate from 7.00 to 8.00. A similar relationship was also observed by Grabowski et al.^54^ who indicated that turf with a high proportion of perennial ryegrass had the worst winter survival rate, which was not confirmed in the present study. This might mean that not only species, but also their varieties could vary in their ability to survive winter.

## Conclusions

The conducted experiment showed that the composed grass mixtures based on the dominance of meadow grass and perennial ryegrass are characterized by higher quality and usefulness for football fields compared to commercial grass mixtures based on red fescue, which confirmed our hypothesis.

In general, we observed that the values of the 6 tested elements (general visual aspect, turf, colour, leaf blade width, susceptibility to diseases, overwintering) statistically differ depending on the year of the research and the tested mixtures. It is worth noting, however, that the composed mixtures were characterized by averagely higher values of the tested parameters than commercial mixtures.

The experiment showed that the mixtures of M7, M8, M11 and M12 grasses are of the highest quality and suitability for football fields sowing. Compared to other mixtures, the M7 and M8 mixtures based on the dominance of meadow grass, were characterized by higher values of the general visual aspect, colour and slenderness of the leaf blade. On the other hand, in terms of wintering, resistance to diseases and sodding, the most useful were mixtures M11 and M12, based on the dominance of perennial ryegrass and co-dominance of perennial ryegrass and meadow grass.

## Supplementary Information

Supplementary Information.
