# Supplementary material for: The influence of the grass mixture composition on the quality and suitability for football pitches
Source: Sci Rep. 2021 Oct 18;11:20592. doi: 10.1038/s41598-021-99859-9 (PMC8523543; doi:10.1038/s41598-021-99859-9)
Supplement: Supplementary file 1 — Supplementary Information. [file 41598_2021_99859_MOESM1_ESM.docx]

**Supplementary Data**

**The influence of the grass mixture composition on the quality and suitability for football pitches**

Karol Wolski ^1^, Joanna Markowska ^2^, Adam Radkowski ^3^, Marek Brennensthul ^4^, Łukasz Sobol ^4^, Grzegorz Pęczkowski ^5^, Henryk Bujak ^6,7^, Wiktoria Grzebieniarz ^8^, Iwona Radkowska^9^ and Karen Khachatryan^8*^

^1^ Wroclaw University of Environmental and Life Sciences, Institute of Agroecology and Plant Production, Wroclaw, Poland, ORCID: 0000-0001-9324-4968; karol.wolski@upwr.edu.pl

^2^ Wroclaw University of Environmental and Life Sciences, Institute of Environmental Engineering, Wroclaw, Poland, ORCID: 0000-0001-8032-9508, joanna.markowska@upwr.edu.pl

^3^ University of Agriculture in Kraków, Department of Agroecology and Plant Production, Kraków, Poland, ORCID: 0000-0002-3146-6212; adam.radkowski@urk.edu.pl

^4^ Wroclaw University of Environmental and Life Sciences, Institute of Agricultural Engineering, Wroclaw, Poland, ORCID: 0000-0003-4964-482X, marek.brennensthul@upwr.edu.pl

^5^ Wroclaw University of Environmental and Life Sciences, Institute of Environmental Protection and Management, Wroclaw, Poland, ORCID: 0000-0001-9660-1326; grzegorz.peczkowski@upwr.edu.pl

^6^ Wrocław University of Environmental and Life Sciences, Department of Genetics, Plant Breeding and Seed Production, Wrocław, Poland, ORCID: 0000-0001-8095-2105; henryk.bujak@upwr.edu.pl

^7^ Research Centre for Cultivar Testing in Slupia Wielka, 63-022, Slupia Wielka, Poland, ORCID: 0000-0001-8095-2105

^8^ Faculty of Food Technology, University of Agriculture in Krakow, Balicka Str. 122, 30-149 Krakow, Poland;

ORCID: 0000-0001-7950-0582; wiktoria.grzebieniarz@urk.edu.pl (W.G.)

ORCID: 0000-0001-7823-5406; karen.khachatryan@urk.edu.pl (K.K.)

^9^Department of Cattle Breeding, National Research Institute of Animal Production, Krakowska 1, 32-083 Balice, Poland, ORCID: 0000-0002-8780-1585; iwona.radkowska@iz.edu.pl (I.R.)

*Correspondence: karen.khachatryan@urk.edu.pl (K.K.)

The results of the statistical analysis are presented below; A – Study year, B – Type of the mixture

Table 1. Ratings of pitch overall aspect in spring across grass mixtures and research years

| Mixtures (B) | Year (A) | | | Mean |
| --- | --- | --- | --- | --- |
|  | 2007 | 2008 | 2009 |  |
| M_1_ | 5.34 o | 5.15 p | 5.34 o | 5.29 i |
| M_2_ | 5.52 n | 5.34 o | 5.86 ł | 5.57 f |
| M_3_ | 4.67 t | 5.34 o | 6.50 i | 5.48 g |
| M_4_ | 5.81 m | 7.02 f | 7.51 c | 6.76 c |
| M_5_ | 4.33 w | 4.58 u | 4.00 x | 4.28 k |
| M_6_ | 4.33 w | 4.75 s | 6.00 l | 5.02 j |
| M_7_ | 7.84 b | 6.50 i | 8.01 a | 7.45 a |
| M_8_ | 6.35 j | 6.50 i | 7.02 f | 6.60 d |
| M_9_ | 2.99 z | 6.50 i | 7.18 e | 5.38 h |
| M_10_ | 3.31 y | 6.05 i | 6.66 h | 5.24 i |
| M_11_ | 5.02 r | 6.66 h | 6.00 l | 5.86 e |
| M_12_ | 7.34 d | 6.86 g | 7.02 f | 7.08 b |
| Mean | 5.15 c | 5.90 b | 6.40 a | 5.81 |
| NIR_α=0,05_ dla: A = 0.03 B = 0.07 A x B = 0.13 | | | | |

Table 2. Ratings of pitch overall aspect in summer across grass mixtures and research years

| Mixtures (B) | Year (A) | | | Mean |
| --- | --- | --- | --- | --- |
|  | 2007 | 2008 | 2009 |  |
| M_1_ | 7.18 h | 5.34 r | 7.34 g | 6.60 g |
| M_2_ | 7.18 h | 6.86 j | 7.02 i | 7.02 f |
| M_3_ | 5.02 r | 6.86 j | 6.66 k | 6.15 i |
| M_4_ | 6.00 n | 6.00 n | 7.34 g | 6.45 h |
| M_5_ | 5.86 o | 3.50 u | 4.49 t | 4.54 k |
| M_6_ | 5.86 o | 6.00 n | 5.66 p | 5.86 j |
| M_7_ | 7.51 f | 8.01 d | 8.01 d | 7.84 b |
| M_8_ | 7.67 e | 8.01 d | 8.01 d | 7.90 a |
| M_9_ | 6.50 l | 9.00 a | 7.51 f | 7.62 d |
| M_10_ | 6.66 k | 8.01 d | 8.01 d | 7.56 e |
| M_11_ | 6.35 m | 8.53 b | 8.01 d | 7.56 e |
| M_12_ | 7.67 e | 8.18 c | 7.51 f | 7.78 c |
| Mean | 6.60 c | 6.92 b | 7.08 a | 6.86 |
| NIR_α=0,05_ dla: A = 0.04 B = 0.05 A x B = 0.09 | | | | |

Table 3. Ratings of pitch overall aspect in autumn across grass mixtures and research years

| Mixtures (B) | Year (A) | | | Mean |
| --- | --- | --- | --- | --- |
|  | 2007 | 2008 | 2009 |  |
| M_1_ | 9.00 a | 7.02 h | 6.15 j | 7.34 e |
| M_2_ | 8.64 b | 7.02 h | 6.15 j | 7.24 f |
| M_3_ | 6.50 i | 7.02 h | 6.50 i | 6.66 h |
| M_4_ | 6.15 j | 6.15 j | 6.10 j | 6.15 i |
| M_5_ | 5.02 k | 5.02 k | 2.76 m | 4.20 k |
| M_6_ | 6.50 i | 4.00 l | 7.02 h | 5.76 j |
| M_7_ | 8.18 c | 9.00 a | 8.01 d | 8.41 a |
| M_8_ | 8.18 c | 8.01 d | 7.51 g | 7.90 c |
| M_9_ | 7.51 g | 7.67 f | 6.50 i | 7.24 f |
| M_10_ | 6.50 i | 6.97 h | 8.01 d | 7.13 g |
| M_11_ | 7.84 e | 7.02 h | 7.67 f | 7.51 d |
| M_12_ | 8.53 b | 8.01 d | 8.18 c | 8.24 b |
| Mean | 7.34 a | 6.86 b | 6.60 c | 6.92 |
| NIR_α=0,05_ dla: A = 0.05 B = 0.08 A x B = 0.14 | | | | |

Table 4. Ratings of pitch density in spring across grass mixtures and research years

| Mixtures (B) | Year (A) | | | Mean |
| --- | --- | --- | --- | --- |
|  | 2007 | 2008 | 2009 |  |
| M_1_ | 4.84 n | 6.15 j | 6.15 j | 5.71 e |
| M_2_ | 4.84 n | 6.15 j | 6.15 j | 5.71 e |
| M_3_ | 3.65 p | 6.35 i | 6.50 h | 5.43 f |
| M_4_ | 4.97 mn | 7.18 e | 6.86 g | 6.30 d |
| M_5_ | 4.97 mn | 5.02 m | 5.34 l | 5.11 g |
| M_6_ | 3.50 r | 5.34 l | 5.02 m | 4.58 h |
| M_7_ | 7.51 c | 7.67 b | 7.84 a | 7.67 a |
| M_8_ | 5.66 k | 7.18 e | 7.34 d | 6.71 c |
| M_9_ | 1.96 t | 6.86 g | 7.67 b | 5.11 g |
| M_10_ | 2.96 s | 7.67 b | 7.18 e | 5.71 e |
| M_11_ | 4.49 o | 7.51 c | 7.02 f | 6.25 d |
| M_12_ | 6.50 g | 7.51 c | 6.50 h | 6.81 b |
| Mean | 4.54 b | 6.66 a | 6.60 a | 5.90 |
| NIR_α=0,05_ dla: A = 0.07 B = 0.07 A x B = 0.14 | | | | |

Table 5. Ratings of pitch density in summer across grass mixtures and research years

| Mixtures (B) | Year (A) | | | Mean |
| --- | --- | --- | --- | --- |
|  | 2007 | 2008 | 2009 |  |
| M_1_ | 6.66 j | 4.67 t | 6.86 i | 6.00 g |
| M_2_ | 6.66 j | 5.52 p | 7.02 h | 6.40 f |
| M_3_ | 5.66 o | 7.02 h | 6.50 k | 6.40 f |
| M_4_ | 6.00 n | 7.51 e | 6.35 l | 6.60 e |
| M_5_ | 6.15 m | 4.84 s | 6.86 i | 5.90 h |
| M_6_ | 5.52 p | 6.00 n | 5.02 r | 5.52 i |
| M_7_ | 7.51 e | 8.18 b | 7.18 g | 7.62 a |
| M_8_ | 7.18 g | 7.84 d | 7.51 e | 7.51 b |
| M_9_ | 5.52 p | 8.35 a | 7.02 h | 6.92 d |
| M_10_ | 6.50 k | 8.18 b | 7.34 f | 7.34 c |
| M_11_ | 6.86 i | 8.01 c | 8.01 c | 7.62 a |
| M_12_ | 7.51 e | 7.51 e | 7.02 h | 7.34 c |
| Mean | 6.45 c | 6.92 a | 6.86 b | 6.76 |
| NIR_α=0,05_ dla: A = 0.03 B = 0.05 A x B = 0.09 | | | | |

Table 6. Ratings of pitch density in autumn across grass mixtures and research years

| Mixtures (B) | Year (A) | | | Mean |
| --- | --- | --- | --- | --- |
|  | 2007 | 2008 | 2009 |  |
| M_1_ | 8.53 d | 8.01 f | 7.67 h | 8.07 b |
| M_2_ | 8.82 b | 8.01 f | 7.67 h | 8.18 a |
| M_3_ | 7.51 i | 7.02 l | 7.51 i | 7.34 f |
| M_4_ | 7.34 j | 7.51 i | 7.84 g | 7.56 e |
| M_5_ | 5.81 o | 7.02 l | 5.06 p | 5.95 h |
| M_6_ | 7.51 i | 5.02 p | 6.15 n | 6.20 g |
| M_7_ | 8.64 c | 7.84 g | 7.51 i | 8.01 c |
| M_8_ | 8.18 e | 7.51 i | 7.02 l | 7.56 e |
| M_9_ | 8.53 d | 8.01 f | 7.18 k | 7.90 d |
| M_10_ | 7.84 g | 7.84 g | 8.01 f | 7.90 d |
| M_11_ | 7.84 g | 7.02 l | 7.67 h | 7.51 e |
| M_12_ | 9.00 a | 7.18 k | 6.50 m | 7.51 e |
| Mean | 7.95 a | 7.29 b | 7.13 c | 7.45 |
| NIR_α=0,05_ dla: A = 0.05 B = 0.05 A x B = 0.09 | | | | |

Table 7. Ratings of pitch colour across grass mixtures and research years in spring

| Mixtures (B) | Year (A) | | | Mean |
| --- | --- | --- | --- | --- |
|  | 2007 | 2008 | 2009 |  |
| M_1_ | 7.02 b | 4.49 f | 7.02 b | 6.10 f |
| M_2_ | 8.01 a | 7.02 b | 7.02 b | 7.34 a |
| M_3_ | 8.01 a | 7.02 b | 7.02 b | 7.34 a |
| M_4_ | 7.02 b | 5.66 d | 7.02 b | 6.55 d |
| M_5_ | 6.00 c | 5.57 d | 8.01 a | 6.50 d |
| M_6_ | 7.02 b | 5.02 e | 7.02 b | 6.30 e |
| M_7_ | 8.01 a | 6.15 c | 7.02 b | 7.02 b |
| M_8_ | 7.02 b | 6.00 c | 7.02 b | 6.66 c |
| M_9_ | 7.02 b | 6.00 c | 7.02 b | 6.66 c |
| M_10_ | 7.02 b | 5.62 d | 7.02 b | 6.55 d |
| M_11_ | 6.00 c | 5.15 e | 6.00 c | 5.71 g |
| M_12_ | 7.02 b | 6.00 c | 7.02 b | 6.66 c |
| Mean | 7.08 a | 5.76 c | 7.02 b | 6.60 |
| NIR_α=0,05_ dla: A = 0.05 B= 0.10 A x B = 0.18 | | | | |

Table 8. Ratings of pitch colour across grass mixtures and research years in summer

| Mixtures (B) | Year (A) | | | Mean |
| --- | --- | --- | --- | --- |
|  | 2007 | 2008 | 2009 |  |
| M_1_ | 7.02 c | 4.97 f | 4.00 g | 5.24 f |
| M_2_ | 7.02 c | 7.02 c | 7.02 c | 7.02 d |
| M_3_ | 8.01 a | 7.02 c | 7.02 c | 7.34 b |
| M_4_ | 6.00 e | 6.50 d | 7.02 c | 6.50 e |
| M_5_ | 7.02 c | 1.99 h | 5.02 f | 4.41 g |
| M_6_ | 7.02 c | 7.02 c | 7.02 c | 7.02 d |
| M_7_ | 7.02 c | 7.02 c | 8.01 a | 7.34 b |
| M_8_ | 8.01 a | 8.01 a | 7.02 c | 7.67 a |
| M_9_ | 6.00 | 7.02 c | 6.45 d | 6.50 e |
| M_10_ | 8.01 a | 7.02 c | 7.02 c | 7.34 b |
| M_11_ | 7.02 c | 8.01 a | 7.02 c | 7.34 b |
| M_12_ | 8.01 a | 7.51 b | 6.00 e | 7.13 c |
| Mean | 7.18 a | 6.45 c | 6.50 b | 6.71 |
| NIR_α=0,05_ dla: A = 0.02 B = 0.03 A x B = 0.05 | | | | |

Table 9. Ratings of pitch colour across grass mixtures and research years in autumn

| Mixtures (B) | Year (A) | | | Mean |
| --- | --- | --- | --- | --- |
|  | 2007 | 2008 | 2009 |  |
| M_1_ | 7,02 d | 5,02 f | 7,02 d | 6,30 f |
| M_2_ | 8,01 a | 5,02 f | 7,02 d | 6,60 e |
| M_3_ | 7,02 d | 5,02 f | 7,02 d | 6,30 f |
| M_4_ | 7,02 d | 6,00 e | 7,02 d | 6,66 d |
| M_5_ | 4,00 g | 1,99 h | 7,02 d | 4,08 j |
| M_6_ | 8,01 a | 1,99 h | 7,84 b | 5,52 i |
| M_7_ | 7,02 d | 6,00 e | 8,01 a | 6,97 c |
| M_8_ | 8,01 a | 6,00 e | 8,01 a | 7,29 a |
| M_9_ | 6,00 e | 6,00 e | 6,00 e | 6,00 h |
| M_10_ | 4,00 g | 7,02 d | 7,67 c | 6,10 g |
| M_11_ | 4,00 g | 7,02 d | 7,67 c | 6,10 g |
| M_12_ | 7,02 d | 7,02 d | 7,67 c | 7,24 b |
| Mean | 6,35 b | 5,15 c | 7,34 a | 6,25 |
| NIR_α=0,05_ dla: A = 0.02 B = 0.02 A x B = 0.03 | | | | |

Table 10. Leaf fineness in spring across grass mixtures and research years

| Mixtures (B) | Year (A) | | | Mean |
| --- | --- | --- | --- | --- |
|  | 2007 | 2008 | 2009 |  |
| M_1_ | 5.81 l | 6.00 k | 5.02 o | 5.62 j |
| M_2_ | 7.51 c | 6.86 g | 6.00 k | 6.76 fg |
| M_3_ | 7.51 c | 7.02 f | 6.00 k | 6.81 f |
| M_4_ | 7.67 b | 7.84 a | 7.02 f | 7.51 a |
| M_5_ | 7.67 b | 6.00 k | 6.00 k | 6.55 h |
| M_6_ | 7.02 f | 5.66 m | 6.00 k | 6.20 i |
| M_7_ | 6.35 j | 7.67 b | 7.02 f | 7.02 d |
| M_8_ | 7.02 f | 7.34 d | 7.02 f | 7.13 c |
| M_9_ | 7.67 b | 7.18 e | 7.02 f | 7.29 b |
| M_10_ | 6.50 i | 6.66 h | 7.02 f | 6.71 g |
| M_11_ | 6.66 h | 7.02 f | 7.02 f | 6.92 e |
| M_12_ | 6.35 j | 7.02 f | 5.20 n | 6.15 i |
| Mean | 6.97 a | 6.86 b | 6.35 c | 6.71 |
| NIR_α=0,05_ dla: A = 0.04 B = 0.06 A x B = 0.10 | | | | |

Table 11. Leaf fineness in summer across grass mixtures and research years

| Mixtures (B) | Year (A) | | | Mean |
| --- | --- | --- | --- | --- |
|  | 2007 | 2008 | 2009 |  |
| M_1_ | 7.02 g | 6.00 j | 5.02 l | 6.00 l |
| M_2_ | 7.34 e | 6.35 i | 6.00 j | 6.55 j |
| M_3_ | 7.51 d | 7.02 g | 6.00 j | 6.81 g |
| M_4_ | 7.84 b | 7.34 e | 7.02 g | 7.40 c |
| M_5_ | 7.51 d | 6.35 i | 6.35 i | 6.71 i |
| M_6_ | 6.86 h | 6.00 j | 5.52 k | 6.10 k |
| M_7_ | 8.01 a | 8.01 a | 8.01 a | 8.01 a |
| M_8_ | 7.18 f | 7.02 g | 7.02 g | 7.08 f |
| M_9_ | 7.34 e | 7.02 g | 7.02 g | 7.13 e |
| M_10_ | 7.34 e | 7.02 g | 6.00 j | 6.76 h |
| M_11_ | 7.84 b | 7.67 c | 8.01 a | 7.84 b |
| M_12_ | 7.84 b | 7.02 g | 7.02 g | 7.29 d |
| Mean | 7.45 a | 6.86 b | 6.55 c | 6.97 |
| NIR_α=0.05_ dla: A = 0.03 B = 0.04 A x B = 0.08 | | | | |

Table 12. Leaf fineness in autumn across grass mixtures and research years

| Mixtures (B) | Year (A) | | | Mean |
| --- | --- | --- | --- | --- |
|  | 2007 | 2008 | 2009 |  |
| M_1_ | 6.35 i | 5.52 m | 5.34 n | 5.71 i |
| M_2_ | 7.67 c | 7.34 d | 7.02 e | 7.34 b |
| M_3_ | 7.34 d | 6.30 i | 7.02 e | 6.86 e |
| M_4_ | 7.34 d | 6.66 g | 7.02 e | 7.02 cd |
| M_5_ | 6.66 g | 6.15 j | 5.02 o | 5.95 h |
| M_6_ | 5.34 n | 5.34 n | 5.02 o | 5.24 j |
| M_7_ | 6.86 f | 7.02 e | 7.02 e | 6.97 d |
| M_8_ | 7.84 b | 8.01 a | 7.02 e | 7.62 a |
| M_9_ | 7.34 d | 8.01 a | 6.00 k | 7.08 c |
| M_10_ | 6.35 i | 6.00 k | 5.34 n | 5.90 h |
| M_11_ | 6.86 f | 6.00 k | 6.50 h | 6.45 f |
| M_12_ | 6.66 g | 6.00 k | 5.66 l | 6.10 g |
| Mean | 6.86 a | 6.50 b | 6.15 c | 6.50 |
| NIR_α=0.05_ dla: A = 0.05 B = 0.06 A x B = 0.10 | | | | |

Table 13. Ratings of winter survival across grass mixtures and research years

| Mixtures (B) | Year (A) | | | Mean |
| --- | --- | --- | --- | --- |
|  | 2007/2008 | 2008/2009 | 2009/2010 |  |
| M_1_ | 6.50 o | 7.18 k | 6.35 p | 6.66 i |
| M_2_ | 6.30 p | 6.81 m | 6.86 m | 6.66 i |
| M_3_ | 7.62 h | 7.84 g | 7.34 j | 7.62 d |
| M_4_ | 8.82 b | 8.18 e | 7.51 hi | 8.18 b |
| M_5_ | 7.45 ij | 8.35 d | 6.66 n | 7.45 ef |
| M_6_ | 6.10 r | 8.82 b | 7.02 l | 7.29 g |
| M_7_ | 8.01 f | 6.00 s | 7.02 l | 6.97 h |
| M_8_ | 8.01 f | 6.35 p | 7.84 g | 7.40 f |
| M_9_ | 7.34 j | 7.34 j | 7.84 g | 7.51 e |
| M_10_ | 8.53 c | 7.45 ij | 8.18 e | 8.07 c |
| M_11_ | 8.64 c | 8.53 c | 8.53 c | 8.53 a |
| M_12_ | 7.51 hi | 9.00 a | 9.00 a | 8.47 a |
| Mean | 7.56 b | 7.62 a | 7.51 c | 7.56 |
| NIR_α=0,05_ dla: A = 0.04 B = 0.08 A x B = 0.13 | | | | |
